# Perioperative outcomes of robotic surgery for the treatment of lung cancer compared to a conventional video-assisted thoracoscopic surgery (VATS) technique

**DOI:** 10.18632/oncotarget.19533

**Published:** 2017-07-25

**Authors:** Zipu Yu, Qiong Xie, Lei Guo, Xin Chen, Chenyao Ni, Wenzong Luo, Weidong Li, Liang Ma

**Affiliations:** ^1^Department of Thoracic Surgery, 2nd Affiliated Hospital, Zhejiang University, Hangzhou, China; ^2^Department of Cardiothoracic Surgery, 1st Affiliated Hospital, Zhejiang University, Hangzhou, China

**Keywords:** robotic, video-assisted thoracoscopic surgery, lung cancer, da Vinci robotic system, meta-analysis

## Abstract

**Aim:**

To conduct a meta-analysis to determine the relative merits between robotic video-assisted thoracoscopic surgery (R-VATS) and conventional video-assisted thoracoscopic surgery (VATS) for lung cancer.

**Results:**

Fifteen studies matched the selection criterion, which reported 8827 subjects, of whom 1704 underwent R-VATS and 7123 underwent VATS. Compared the perioperative outcomes with VATS, reports of R-VATS indicated unfavorable outcomes considering the operative time (SMD = 0.48, 95% CI 0.15 to 0.81). Meanwhile, the number of dissected lymph nodes (SMD = 0.12, 95% CI −0.27 to 0.51) and hospital stay following surgery (SMD = −0.1; 95% CI −0.27 to 0.07), conversion (RR = 0.68; 95% CI 0.42 to 1.11), morbidity (RR = 0.99, 95% CI 0.92 to 1.07) and mortality (RR = 0.33, 95% CI 0.1 to 1.09) were similar for both procedures.

**Materials and Methods:**

A literature search was performed to identify comparative studies reporting perioperative outcomes for R-VATS and VATS for lung cancer. Pooled risk ratio (RR) and standardized mean differences (SMDs) with 95% confidence intervals (95% CIs) were calculated using either the fixed effects model or the random effects model.

**Conclusions:**

There is no difference in terms of perioperative outcomes between R-VATS and VATS except for the operative time which is significantly high for R-VATS. Further studies are required to confirm these results.

## INTRODUCTION

The introduction of minimally invasive surgery (MIS) has opened new possibilities in various surgical fields. Benefits of video-assisted thoracic surgery (VATS) have been reported for its shorter length of hospital stay, decreased pain, a more rapid return to normal activity [1–3]. Video-assisted thoracoscopic surgery (VATS) is widely accepted as a safe and useful approach for the management of various thoracic conditions [4]. However, VATS still remains a technically challenging procedure owing to its two-dimensional visual representation and use of nonflexible endoscopic instruments.

Robotic surgery was introduced as an evolution of video-assisted thoracic surgery while maintaining advantages in part to overcome the limitations of VATS at the end of 1990s. The robotic approach has many advantages, which include greater flexibility and higher definition three-dimensional vision, more intuitive movements and comfort of the surgeon via the use of wrist instruments [5, 6]. Probably the first series using a robotic system to perform lung lobectomy was published in 2002 [7]. As a whole, robotic surgery still remains in its infancy.

Systematic review and meta-analysis is an important tool for revealing trends that might not be apparent in a single study. Pooling of independent but similar studies increases precision and confidence [8]. In this study, we aimed to determine the relative merits of R-VATS and VATS for lung cancer.

## RESULTS

### Description of the included studies

**Figure 1:**
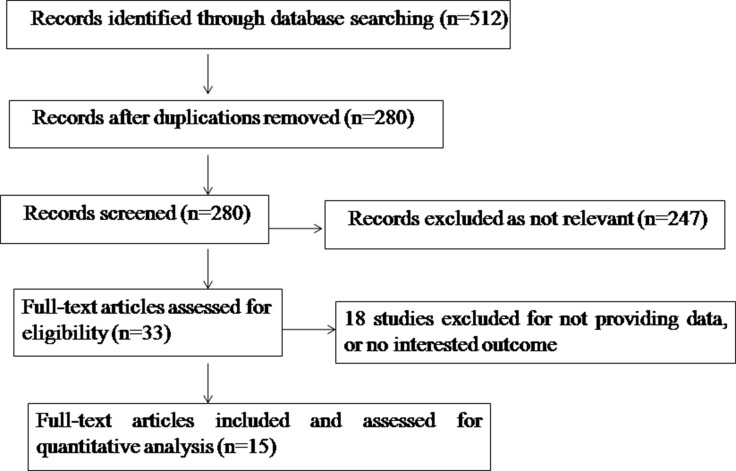
Flow chart indicating the process of selecting articles for meta-analysis

The initial search strategy retrieved 280 publications after removing duplications. Overall, 15 studies [9–23] met our entry criteria and were included in the analysis. A flow diagram of the study selection process is presented in Figure 1. All characteristics of studies are summarized in Table 1.

**Table 1:** Characteristics of included studies

Study	Author	Year	Country	Design	Study quality	Group	Patients in each group, *n*
1	R.Douglas Adams et al.	2014	USA	ROS	4/9	R-VATS/ VATS	116/4612
2	Yong He et al.	2014	UK	POS	7/9	R-VATS/ VATS	30/34
3	Julien Mahieu et al.	2015	France	ROS	5/9	R-VATS/ VATS	28/28
4	Benjamin E. Lee et al.	2015	USA	ROS	5/9	R-VATS/ VATS	53/158
5	Hyun-Sung Lee et al.	2012	Korea	ROS	5/9	R-VATS/ VATS	100/100
6	Michael Kent et al.	2014	USA	ROS	6/9	R-VATS/ VATS	411/1233
7	Florian Augustin et al.	2013	Austria	ROS	7/9	R-VATS/ VATS	26/26
8	Brian E. Louie et al.	2012	USA	ROS	6/9	R-VATS/ VATS	46/34
9	Scott J. Swanson et al.	2014	USA	ROS	6/9	R-VATS/ VATS	295 + 325/295 + 325
10	Hee-Jin Jang et al.	2011	Korea	ROS	6/9	R-VATS/ VATS	40/40
11	Shaun A. Deen et al.	2014	USA	ROS	5/9	R-VATS/ VATS	57/58
12	Adalet Demir et al.	2015	Turkey	ROS	6/9	R-VATS/ VATS	34/65
13	Benjamin E. Lee et al.	2014	USA	ROS	7/9	R-VATS/ VATS	35/34
14	Benedetto Mungo et al.	2016	USA	ROS	5/9	R-VATS/ VATS	80/53
15	Julien Mahieu et al.	2016	France	ROS	4/9	R-VATS/ VATS	28/28

ROS: retrospective observational study; POS: prospective observational study; VATS: video-assisted thoracoscopic surgery; R-VATS: robotic video-assisted thoracoscopic surgery.

### Meta-analysis of intra-operative data

**Figure 2:**
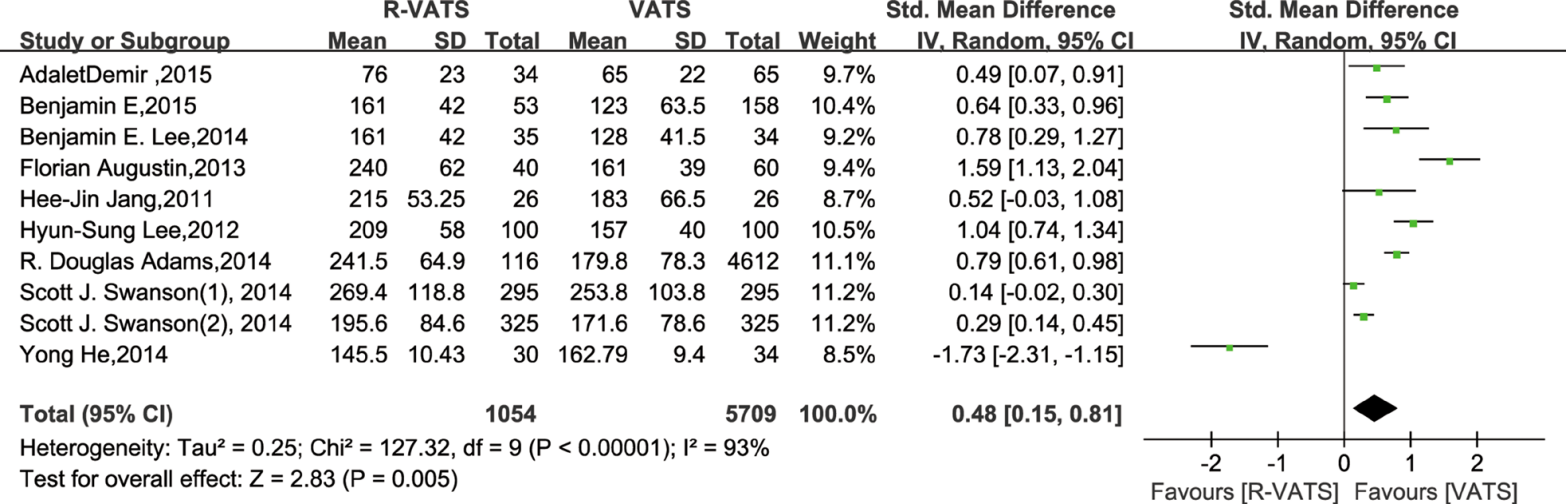
Forest plot presenting operating time from the studies included 95% CI: 95% confidence interval.

The random-effects meta-analysis results indicated that the operating time was significantly different between R- VATS and VATS (SMD, 0.48; 95% CI, 0.15–0.81; *P* = 0.005). The ten sets of results showed a significant amount of heterogeneity (*I*^2^ = 93%, *P* < 0.00001) (Figure 2).

**Figure 3:**
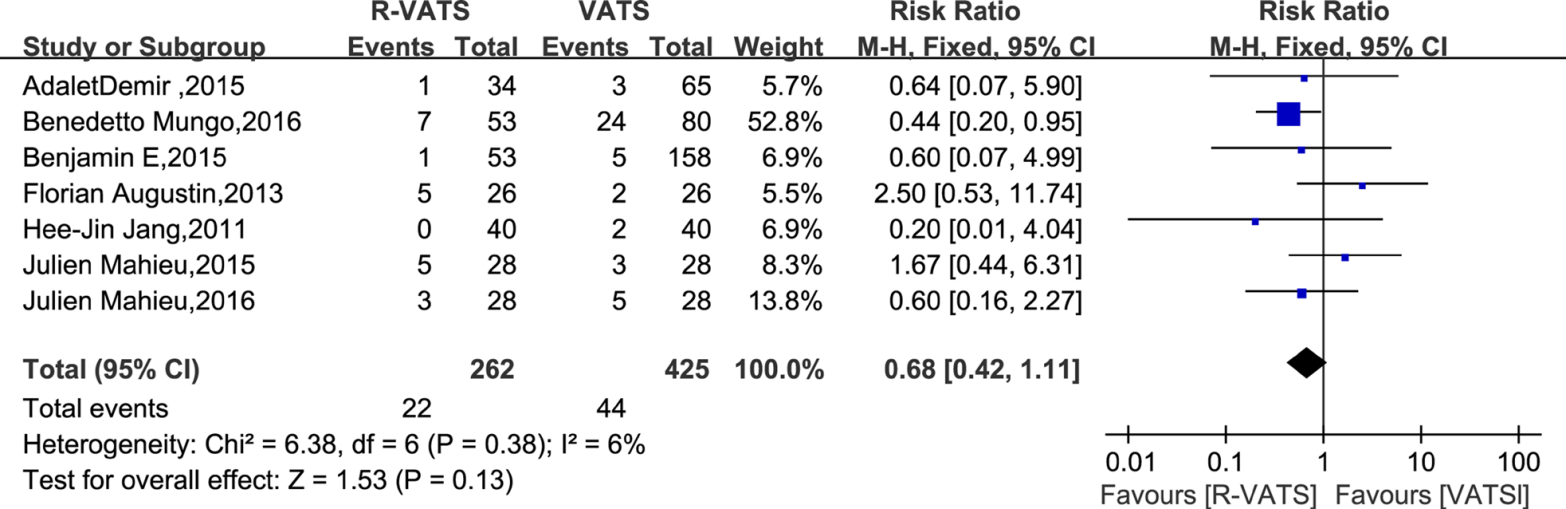
Forest plot presenting conversion from the studies included 95% CI: 95% confidence interval.

Seven studies reported on conversion. Conversion is adopted for the reason that it is difficult to perform operations as planned for R-VATS. Conversion of R-VATS is carried out by the way of using a rib-spreading thoracotomy or switching from robotic to conventional VATS. There was no significant difference between two groups (RR = 0.68; 95% CI 0.42 to 1.11; *P* = 0.13). There was no significant heterogeneity between the studies (*I*^2^ = 0%) (Figure 3).

### Meta-analysis of pathologic details

**Figure 4:**
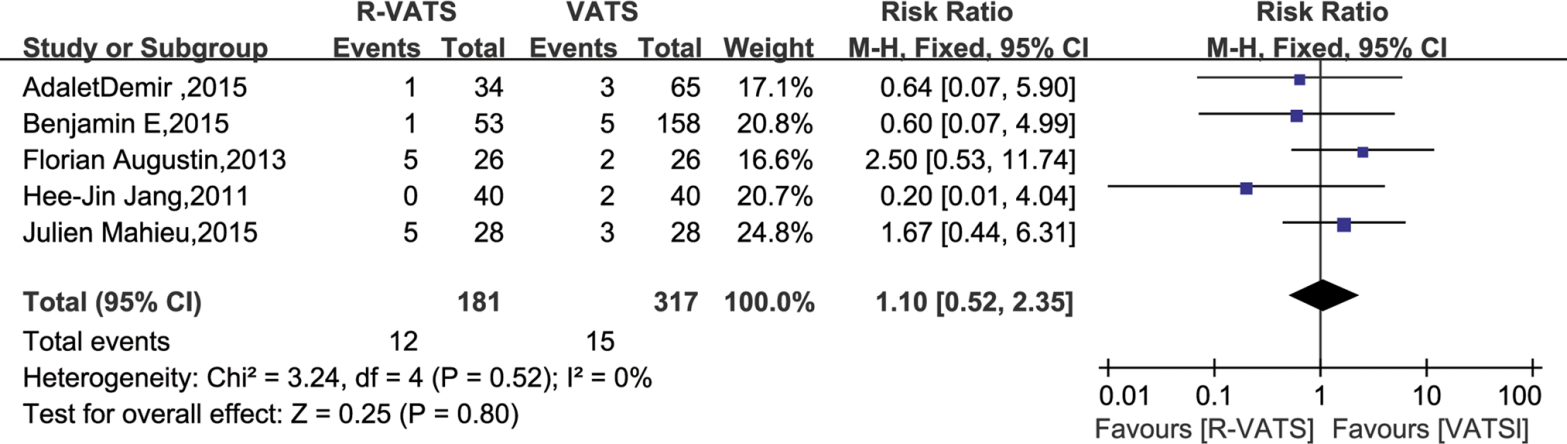
Forest plot presenting the number of dissected lymph nodes from the studies included 95% CI: 95% confidence interval.

In the five studies, there was no significant difference between two groups in the number of lymph nodes harvested (SMD = 0.12, 95% CI −0.27 to 0.51). The random-effects model was used because of the heterogeneity between the studies (*I*^2^ = 78%) (Figure 4).

### Meta-analysis of post-operative outcomes

**Figure 5:**
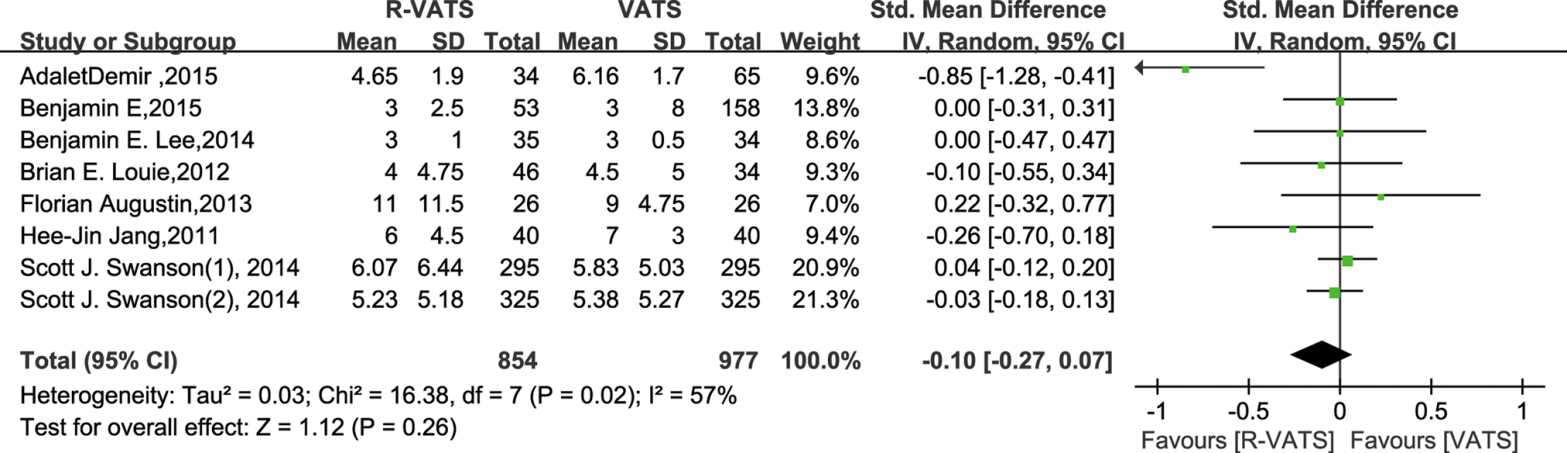
Forest plot presenting length of hospital stay from the studies included 95% CI: 95% confidence interval.

In eight studies, length of hospital stay was found to be no significantly different between the R- VATS and VATS group. Meanwhile, analysis of the pooled data revealed that the two groups did not differ significantly in this regard (SMD: −0.10; 95% CI: −0.27 to 0.07; *P* = 0.26) (Figure 5).

**Figure 6:**
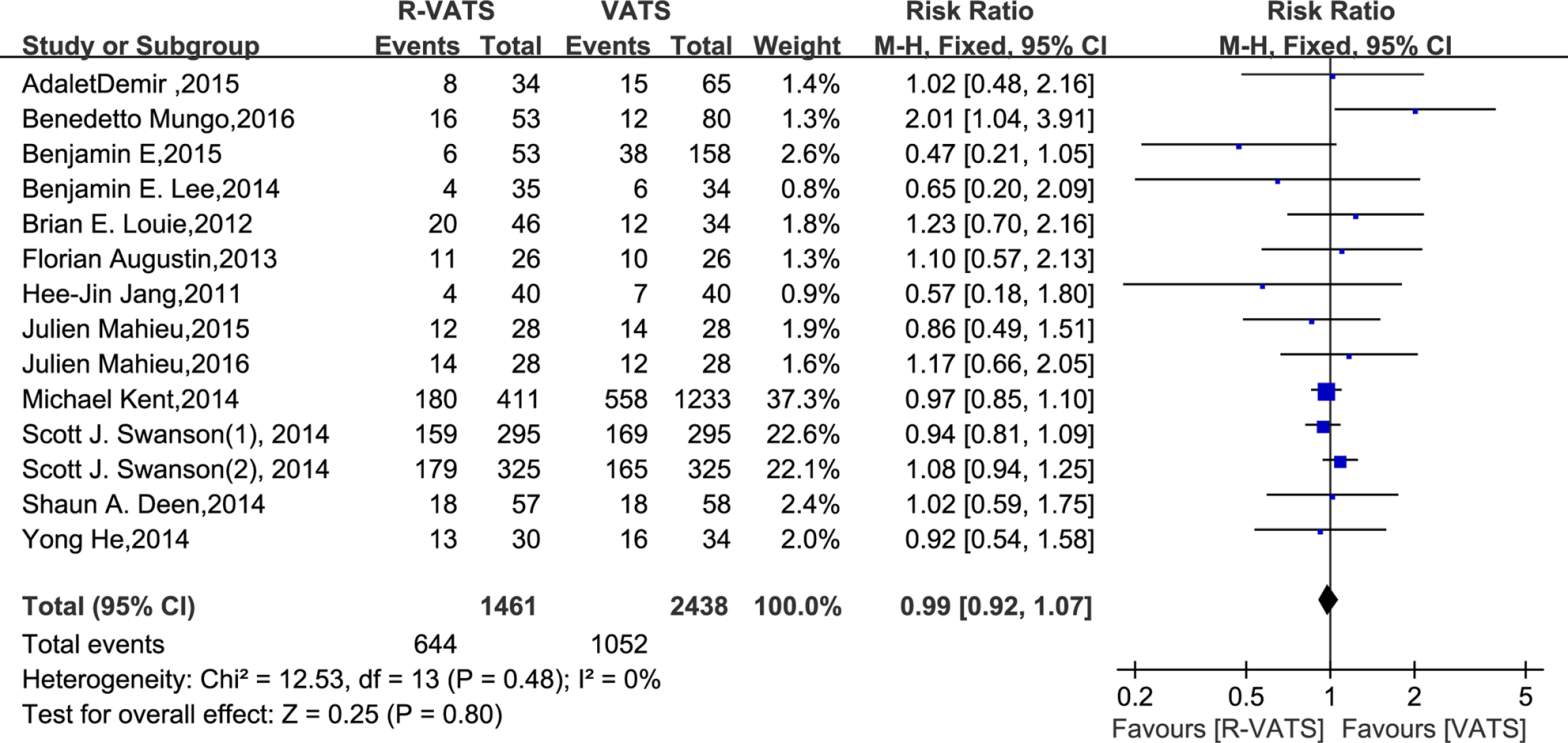
Forest plot presenting morbidity from the studies included 95% CI: 95% confidence interval.

In all fourteen studies, morbidity was found to be no significantly different between the R- VATS and VATS group. Meanwhile, analysis of the pooled data indicated that the two groups did not differ significantly in this regard (RR = 0.99, 95% CI 0.92 to 1.07; *P* = 0.80) (Figure 6).

**Figure 7:**
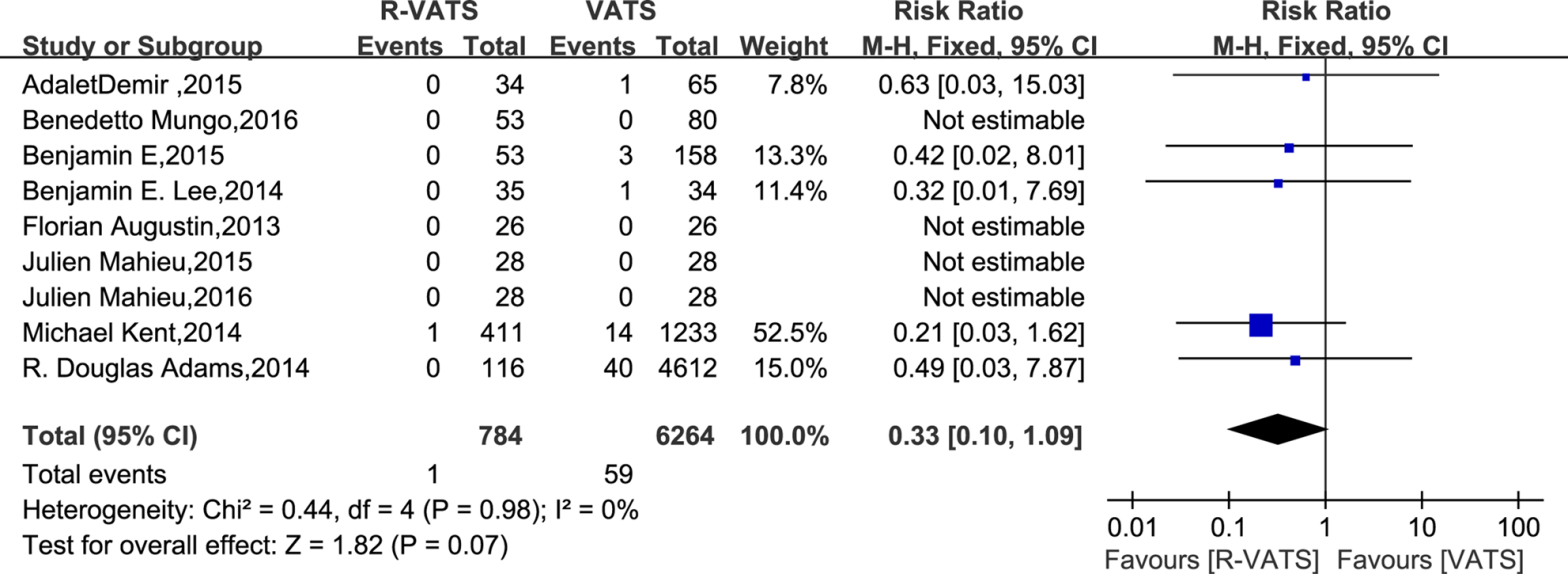
Forest plot presenting in-hospital mortality from the studies included 95% CI: 95% confidence interval.

In nine studies, mortality was found to be no significantly different between the R- VATS and VATS group. Meanwhile, analysis of the pooled data demonstrated that the two groups did not differ significantly in this regard (RR: 0.33, 95% CI: 0.10 to 1.09; *P* = 0.07) (Figure 7).

## DISCUSSION

Traditionally, randomized controlled trials (RCTs) has been used in meta-analysis. However, using non-randomized controlled trials (NRCTs) might be a good method in meta-analysis of some clinical settings in which either the number or sample size of the RCTs is insufficient [24, 25].

In this meta-analysis, we found that there was a significant difference in operating time between R-VATS and VATS. Only one study of Yong He suggested that R-VATS was associated with a shorter time for operating time, compared with VATS approach. Whereas others suggested that operating time was shorter for VATS approach. This may be attributable to the additional set-up time required for R-VATS [26]. With increasing experience and set-up time gradually decreased, the actual time may be shorter in R-VATS. The technical advantage of R-VATS in the thoracic cavity will be more evident compared to the common VATS in the future. Although R-VATS offers a number of advantages over VATS, the results of our meta-analysis suggest that there are no additional clinical benefits for R-VATS over VATS. There were no significant differences in conversion, the number of lymph-node dissection, length of hospital stay, morbidity and mortality between R-VATS and VATS. From the point of clinical practice and patients, these parameters should be taken into account when deciding whether R-VATS technique is superior to the VATS technique.

Whether the R-VATS or VATS approach brings in more advantages remains a matter of debate.

Some studies have suggested that R-VATS was associated with a higher rate of intraoperative conversion, compared with VATS approach. Whereas others have suggested that conversion is comparable between the two approaches. In our analysis study, there was no difference in conversion rate between the R-VATS and VATS approaches. We observed that conversion rate was comparable in the two approaches. This is likely the result of more exquisite skills of surgeons and complete exposure of operation field. Owing to the advantages of three-dimensional optics, the stable camera platform and the flexible instrumentation, one potential strength of the robotic approach might be the thoroughness of the lymphadenectomy. Studies have suggested the robotic approach resulted in more samples of lymph nodes than the VATS approach. However, there was no difference in numbers of lymph nodes sampled between the R-VATS and VATS approaches in the current study. This is likely attributable to the operation subject scale differences and different pathological types for lung cancer from other studies.

Lobectomy is the main procedure type in these studies. Segmentectomy is included in studies of Deen SA and Demir A, while wedge resection is exsit in the study of Swanson SJ. Surgical resection maintains an important role in the treatment of lung carcinoma, so efforts must be directed towards determining methods to reduce morbidity and mortality to achieve optimal pulmonary dynamics in the perioperative periods, which will make a difference in the matter of length of hospital stay. Pulmonary morbidity is a major cause of mortality in patients with lung cancer. Previous series have reported that most of deaths to be directly related to peri-operative morbidity, in particular respiratory failure and pneumonia. In this meta-analysis, there were no significant differences between two groups for morbidity, leading to a similar result for the comparisons of overall mortality rate between two groups. The main direct causes of peri-operative mortality include hemorrhage, respiratory failure, pneumonia and myocardial event. Cardiac and infections are the main sources for morbidity, while the prolonged air leak and bronchopleural fistula also exsit for some cases.

Lung cancer is the leading cause of cancer death. Lung surgery has evolved in the past decades for the purpose of diagnosis or treatment. There are several different minimally invasive modalities accepted for lung cancer, such as robotic thoracic surgery(RTS) and video-assisted thoracic surgery(VATS) [17, 19]. Before the introduction of video-assisted thoracic surgery (VATS), lobectomy for lung cancer required thoracotomy and rib spreading [15, 27–29]. VATS is a much less traumatic approach than thoracotomy, resulting in less pain, shorter hospital stay and other advantages [30–33]. In 2000, the United States FDA approved the Da Vinci surgical robot system for clinical application. Application of the robotic surgical system has opened up a new era, with minimally invasive surgery now elevated to a new stage. The robotic surgery system has been widely used in urinary tract, hepatobiliary, cardiovascular and gynecological surgery [34–37]. Robot-assisted surgery is also being adopted in thoracic oncology and several types of mediastinal and lung resection [6, 38–41].

Owing to its limitations, only 15% to 30% of all thoracic operations were performed by VATS [42]. Since the introduction of the robotic system, a very wide range of attention has been paid to its use across the world. Despite the extensive experience in various fields of surgery, there is little evidence of superiority of R-VATS over VATS. Well-designed and adequately powered, blinded, randomized controlled trials are scarce and the risk for publication bias is significant [43, 44]. The results of our meta-analysis are consistent with the previous studies [15, 16, 18], which indicated that the robotic approach had comparable perioperative outcomes, but did not increase clinical benefits for patients.

However, the results of our meta-analysis should be interpreted with caution because of several limitations. First, data came from NRCTs, which might weaken the quality of the results. Second, reports in languages other than English were excluded, leading to potential bias. Third, types of lung cancer resection were not elaborated in studies included, the number of harvested number of lymph nodes may not be a reliable data in the circumstance of VATS technique. The accuracy in numbers of lymph nodes sampled for robotic resection appears to be more approximate to thoracotomy data when analyzed by clinical T stage [45]. Finally, patients’ baseline characteristics differed between studies, and there was inevitably some variability in the surgical techniques and skills of surgeons. All surgeons need a period of time for the learning curve phase for a technique. Only in recent years, robotic technique has been increasingly used. Nevertheless, since there are no clear standards on the surgical procedures for robotic procedures, it would likely be difficult to set up a universal standard for surgeons according to the learning curve phase. Also the favored surgical approach varies dramatically among surgeons, leading to potential bias [46].

The results of this meta-analysis showed that VATS was associated with a shorter operative time. Thus, we suggest that R-VATS is an alternative to VATS for lung cancer resection without no prominent advantages. Further studies are required to confirm these results.

## MATERIALS AND METHODS

### Search strategy

The Pubmed, the Cochrane Library, and the Web of Science were searched systematically for all articles published in English until June 2016 to compare perioperative outcomes of R-VATS and VATS for lung cancer. The terms used for search were:“robotic” and “lung cancer”. Two authors screened results of the literature search, and the reference lists of the included articles were also screened for potential studies. Two authors independently applied the inclusion and exclusion criteria, and any disagreement was resolved by a third reviewer.

### Inclusion and exclusion criteria

For inclusion in the meta-analysis, a study had to fulfill the following criterion: (1) patients with lung cancer diagnosed; (2) compare the outcomes of R-VATS and VATS, regardless of other diseases; (3) report on at least one of the outcome measures mentioned below; and (4) the one of higher quality was included in the analysis if dual (or multiple) studies were reported by the same institution and/or authors.

Abstracts, letters, editorials and expert opinions, reviews without original data, case reports, and studies lacking outcome measures were excluded. The studies or data were also excluded when: 1) it was impossible to extract the appropriate data from the published results; 2) there was overlap between authors or centers; 3) the outcomes and parameters of patients were not clearly reported; (4) studies lacking information on outcomes.

### Outcomes of interest and data extraction

Data abstraction and quality assessment were performed as described previously [47]. Briefly, two reviewers independently extracted the following parameters from each study: 1) first author and year of publication; 2) study population characteristics; 3) number of subjects who underwent each technique; and lastly, 4) intra-operative data, post-operative data, and pathologic details. The following outcomes were used to compare R-VATS and VATS techniques: 1) intra-operative data, which included operating time (min), and conversion; 2) post-operative data, which included hospital stay following surgery (days), morbility and mortality; and 3) pathologic details, which was number of lymph nodes harvested.

In this meta-analysis, patients were matched for operation time, conversion, lymph nodes harvested, length of hospital stay, morbidity and mortality in two groups. Operative time was defined as the time between the initial incision and complete wound closure, and for robotic surgeries it also included time of docking and undocking. Conversion was defined as the need to use a rib-spreading thoracotomy or the need to switch from robotic to conventional VATS. The number of lymph-node dissection was defined as the quantity of lymph-node harvested during surgeries. Length of hospital stay was defined as postoperative days. Morbidity was defined as any postoperative complications. Mortality was defined as any death occurring during initial hospitalization or within 30 days after surgery.

The quality of observational studies was assessed using the Newcastle–Ottawa scale. The Newcastle–Ottawa scale assesses the quality of study based on the following three aspects: (i) the selection of the study cohort (or cases/controls), (ii) the comparability of the cohorts (or cases/controls) and (iii) the outcome assessment for a cohort study, or the determination of the exposure for a case–control study. The quality of randomized trials was assessed using the Jadad scale [48]. The Jadad scale assesses the quality of randomized studies based on the following aspects: randomization, double blinding, withdrawals and dropouts. A score ≥ 3 denotes a high-quality study. The meta-analysis was performed according to the PRISMAguidelines [49].

### Statistical analysis

The statistical analysis was conducted as described previously [47]. The mean and the variance for articles reporting the median, range and the size of the trial were deduced in a way as described in Stela Pudar Hozo,s article [50]. Briefly, Review Manager 5.2 (RevMan 5.2^®^, Nordic Cochrane Center and Copenhagen, Denmark) was used to perform the meta-analysis. The *I*^2^ statistic was used to quantify the statistical heterogeneity of the studies included, and *I*^2^ values of 25–49, 50–74 and ≥ 75% indicate low, moderate and high heterogeneity, respectively. When the *I*^2^ value was > 50%, indicating the presence of variability among the studies, we chose a random-effects models to perform the meta-analysis.

We analyzed dichotomous variables using estimation of risk ratios with a 95% confidence interval (95% CI) and continuous variables using standardized mean difference (SMD) with a 95% CI. Forest plots were used to present the results of the meta-analysis. A *P*-value < 0.05 was considered to be significant.
